# Perfect Storm: COVID-19 Associated Cardiac Injury and Implications for Neurological Disorders

**DOI:** 10.1089/neur.2020.0002

**Published:** 2020-07-23

**Authors:** Catherine Ruth Jutzeler, Thomas Edward Nightingale, Andrei Vasilievich Krassioukov, Matthias Walter

**Affiliations:** ^1^ Department of Biosystems Science and Engineering, ETH Zurich, Basel, Switzerland.; ^2^ SIB Swiss Institute of Bioinformatics, Lausanne, Switzerland.; ^3^ School of Sport, Exercise, and Rehabilitation Sciences, University of Birmingham, Birmingham, United Kingdom.; ^4^ International Collaboration on Repair Discoveries (ICORD), University of British Columbia, Vancouver, British Columbia, Canada.; ^5^ Department of Neuro-Urology, Swiss Paraplegic Center, Nottwil, Switzerland.

**Keywords:** cardiac injury, COVID-19, injury-associated comorbidities, neurological disorders, risk factors, SARS-CoV-2

## Abstract

Coronavirus disease 2019 (COVID-19) can lead to considerable lung damage and even death. Less is known about the effects of COVID-19 on the cardiovascular system. In their recent *JAMA Cardiology* article, Shi and colleagues reported an association between cardiac injury and higher risk of in-hospital mortality in patients with COVID-19. Approximately 20% (82 patients) of the study cohort presented with a cardiac injury. The investigators identified cardiac injury as an independent risk factor of mortality during hospitalization (52% with cardiac injury vs. 5% without cardiac injury, *p* < 0.001). Consequently, their findings are highly relevant for patients with pre-existing cardiovascular and cerebrovascular diseases. Among those are patients with neurological disorders. There is a considerable prevalence of myocardial injury in patients with acute neurological illness, which appears to adversely affect prognosis. Individuals with an underlying neurological disorder are particularly vulnerable to increased cardio-cerebrovascular disease risk due to physical limitations and the pathophysiology of their condition. Thus, we would like to specifically highlight the attention of health care professionals treating patients with pervasive neurological disorders to their potentially elevated risk of poorer COVID-19 related outcomes.

## Introduction

Viral infections, such as coronavirus disease 2019 (COVID-19), can trigger respiratory infections that may lead to considerable lung damage and even death. Less is known about the effects of the severe acute respiratory syndrome coronavirus 2 (SARS-CoV-2), the causative agent of COVID-19, on the cardiovascular system. In their recent *JAMA Cardiology* article, Shi and colleagues shed light on the association between cardiac injury and a higher risk of in-hospital mortality in patients with laboratory-confirmed COVID-19.^1^ The investigators defined cardiac injury as high-sensitivity troponin I (hs-TNI) above the 99th-percentile upper reference limit. For patients to be included in the analysis, laboratory confirmation, medical information, and core examination results were required.

Of 1004 hospitalized patients, only 416 (median age, 64 years; 51% women) were included in the analysis. Overall, fever was the most common symptom (334 patients [80%]) followed by cough (144 patients [35%]) and shortness of breath (117 patients [28%]). Approximately 20% (82 patients) of the study cohort presented with a cardiac injury. The investigators identified cardiac injury as an independent risk factor of mortality during hospitalization (52% with cardiac injury vs. 5% without cardiac injury, *p* < 0.001). Additionally, a greater proportion of patients with cardiac injury required mechanical ventilation (i.e., non-invasive [46 vs. 4%, *p* < 0.001] and invasive [22 vs. 4%, *p* < 0.001]) and in-hospital complications (e.g., acute respiratory distress syndrome [59 vs. 15%, *p* < 0.001], acute kidney injury (9 vs. <1%, *p* < 0.001]) were more common. Lastly, histories of hypertension (60 vs. 23%, *p* < 0.001), coronary heart disease (29 vs. 6%, *p* < 0.001), chronic heart failure (15 vs. 2%, *p* < 0.001), and cerebrovascular disease (16 vs. 3%, *p* < 0.001) were more common among patients with cardiac injury.

Although the study conveys important information regarding the association between cardiac injury and disease outcome in patients with COVID-19, it comes with major limitations that need to be considered when interpreting the results. First, the authors excluded over 588 patients of the initial cohort, of whom 39% were excluded because of missing cardiac biomarkers, including values of hs-TNI and creatine kinase myocardial band. This is problematic as patients with pre-existing cardiovascular conditions are more likely to get tested for cardiac biomarkers, which can lead to a selection bias. Second, patients with cardiac injury were older (median age, 74 vs. 60 years, *p* < 0.001); had higher levels of leukocytes, procalcitonin, C-reactive protein, and creatinine; and had lower levels of lymphocytes and platelets compared with patients without cardiac injury.

Despite these limitations, the study provides important evidence that COVID-19 can have detrimental effects on the heart, even in patients without pre-existing heart conditions. Importantly, these findings are not unique to COVID-19. Other viral infections, such as influenza A (H1N1)^2^ and SARS-CoV-1,^3^ have also been reported to worsen pre-existing cardiovascular diseases and cause new ones in otherwise healthy individuals.

Consequently, the findings of Shi and colleagues are highly relevant for patients with pre-existing cardiovascular and cerebrovascular diseases. Among those are patients with neurological disorders, including individuals with spinal cord injury,^4^ traumatic brain injury,^5^ multiple sclerosis,^6^ Alzheimer's disease,^7^ and Parkinson's disease.^8^

There is a considerable prevalence of myocardial injury in patients with acute neurological illness, which appears to adversely affect prognosis.^9^ Individuals with an underlying neurological disorder are particularly vulnerable to increased cardio-cerebrovascular disease risk due to physical limitations and the pathophysiology of their condition. For example, individuals with spinal cord injury often present with chronic inflammation and a degree of immune impairment, either as a result of reduced physical activity or autonomic nervous system dysfunction causing immunodeficiency through diminished innervation of lymphoid organs or associated endocrine disorders.^10^ Thus, we would like to specifically highlight the attention of health care professionals treating patients with pervasive neurological disorders to their potentially elevated risk of poorer COVID-19 related outcomes. It is of utmost importance that the practice of social distancing is emphasized in these population groups and vulnerable individuals adhere to self-isolation practices to minimize transmission of the disease. These practices are particularly relevant for individuals with neurological disorders given the increased prevalence and constellation of risk factors identified by Shi and colleagues as important for the prognosis of COVID-19 patients.

Conversely, it remains to be seen what impact COVID-19 has on the progression of existing cardiovascular disease in at-risk individuals with neurological disorders, particularly as other viral infections have been shown to worsen pre-existing cardiovascular diseases and cause new ones in otherwise healthy individuals. Therefore, future research is necessary to investigate to what extent testing positive for COVID-19 exacerbates pre-existing conditions or predisposes individuals with neurological disorders to developing new ones.

## Abbreviations Used

COVID-19: coronavirus disease 2019
hs-TNI: high-sensitivity troponin I
SARS-CoV-2: severe acute respiratory syndrome coronavirus 2
